# Bilayer TMDs for Future FETs: Carrier Dynamics and Device Implications

**DOI:** 10.3390/nano15191526

**Published:** 2025-10-05

**Authors:** Shoaib Mansoori, Edward Chen, Massimo Fischetti

**Affiliations:** 1 Department of Materials Science and Engineering, The University of Texas at Dallas, 800 West Campbell Road, Richardson, TX 75080, USA; 2 Taiwan Semiconductor Manufacturing Company Limited (TSMC), Hsinchu Science Park, Hsinchu 300-096, Taiwan; chenjhm@tsmc.com

**Keywords:** bilayer TMD, WS2, WSe2, first principles, DFT, FET, device scaling, dielectric engineering, short-channel effects, device performance

## Abstract

Bilayer transition metal dichalcogenides (TMDs) are promising materials for next-generation field-effect transistors (FETs) due to their atomically thin structure and favorable transport properties. In this study, we employ density functional theory (DFT) to compute the electronic band structures and phonon dispersions of bilayer WS_2_, WSe_2_, and MoS_2_, and the electron-phonon scattering rates using the EPW (electron-phonon Wannier) method. Carrier transport is then investigated within a semiclassical full-band Monte Carlo framework, explicitly including intrinsic electron-phonon scattering, dielectric screening, scattering with hybrid plasmon–phonon interface excitations (IPPs), and scattering with ionized impurities. Freestanding bilayers exhibit the highest mobilities, with hole mobilities reaching 2300 cm^2^/V·s in WS_2_ and 1300 cm^2^/V·s in WSe_2_. Using hBN as the top gate dielectric preserves or slightly enhances mobility, whereas HfO_2_ significantly reduces transport due to stronger IPP and remote phonon scattering. Device-level simulations of double-gate FETs indicate that series resistance strongly limits performance, with optimized WSe_2_ pFETs achieving ON currents of 820 A/m, and a 10% enhancement when hBN replaces HfO_2_. These results show the direct impact of first-principles electronic structure and scattering physics on device-level transport, underscoring the importance of material properties and the dielectric environment in bilayer TMDs.

## 1. Introduction

The semiconductor industry has experienced extraordinary growth, initially driven by silicon, which can form large, single-crystal structures ideally suited to modern electronic manufacturing. Since the 1970s, silicon’s advantageous properties and scalability have enabled continuous advancements, allowing transistor densities to increase exponentially—a trend famously captured by Moore’s Law [1]. The miniaturization of silicon-based devices has reduced costs, boosted speed, and lowered power consumption, making it the foundation of digital technology. However, as silicon transistor dimensions have approached their physical limits, it has become challenging to further downscale without encountering fundamental limitations [2,3]. These issues include short-channel effects, increased leakage currents, and degradation of device reliability, which hinder the performance of ever-smaller silicon transistors [4,5].

Motivated by the need for high-performance devices beyond silicon’s capabilities, researchers have turned to alternative materials [6,7,8,9,10]. Since the early 2000s, the search for novel materials to sustain and surpass silicon’s legacy in both classical and unconventional computing has intensified. These new materials, especially those with lower dimensionality, offer unique physical properties that address the challenges faced by silicon at nanoscale dimensions. One class of promising candidates is two-dimensional (2D) materials [11,12,13], which, unlike bulk materials, are formed from layers just one atom thick. These materials exhibit unique electrostatic control due to their thin nature, allowing for reduced short-channel effects and exceptional gate control.

Among two-dimensional materials, transition metal dichalcogenides (TMDs) [14,15,16] have garnered significant interest. Structurally, TMDs consist of a transition metal layer sandwiched between two layers of chalcogen atoms, with each M-X-M atomic layer forming strong covalent bonds within the layers but weak van der Waals bonds between them. This unique structure enables TMDs to be scaled to atomic thicknesses while maintaining their semiconducting properties, providing a band gap that graphene lacks and allowing for effective switching in field-effect transistors (FETs). The versatility and tunability of TMDs, along with their stable semiconducting properties, make them ideal for future applications in both energy-efficient and high-performance devices.

Bilayer transition metal dichalcogenides (TMDs) offer unique advantages over monolayers for applications in next-generation transistors and electronic devices. They exhibit a higher density of states, enabling greater charge storage, as well as improved carrier mobility for efficient current flow [17,18]. Bilayer structures enhance fabrication yields [19] by protecting the bottom layer during processing, minimizing defects, and providing better stability under environmental stress. In particular, WSe_2_ and WS_2_ have attracted significant attention for pFET applications due to their intrinsically high hole mobility. With their potential to support further device miniaturization and improved performance, bilayer TMDs represent a promising path for advancing both transistor technology and the broader field of nanoelectronics.

To evaluate the transport properties of bilayer TMDs, it is crucial to consider the dielectric environment, as real-world devices often feature these materials supported by substrates and surrounded by gate dielectrics. This work employs a combination of first-principles and semiclassical computational methods to investigate carrier transport in bilayer TMDs. Density functional theory (DFT) and density functional perturbation theory (DFPT), as implemented in Quantum ESPRESSO [20,21], are used to calculate the full-band electronic structures and phonon dispersions, while electron–phonon scattering rates are obtained using the electron–phonon Wannier (EPW) [22,23,24] approach. These first-principles results are incorporated into semiclassical full-band Monte Carlo simulations, solving the Boltzmann transport equation and accounting for intrinsic electron–phonon scattering, screening by the dielectric environment, scattering with the hybrid plasmon/phonon interface excitations, and with ionized impurities. To study the impact of the dielectric environment on device performance, we consider two extremes: a low-k insulator (hBN) and a high-k insulator (HfO_2_). WS_2_ and WSe_2_ were selected for their relatively high hole mobility, making them promising p-FET candidates, though this advantage at 5–10 nm channels is uncertain. Practical factors such as gate leakage, interface traps, doping, and material growth are beyond the scope here, as we focus on intrinsic, best-case performance [25]. By addressing these critical factors, this study provides a robust framework for understanding and optimizing the electronic transport properties of TMDs, helping their integration into next-generation nanoelectronics.

Our discussion is organized as follows: Section 2 outlines the geometry of the double-gate transistor considered and the theoretical framework underlying our study. Section 3 and Section 4 describe the physical models used for the band structure, phonon dispersion, and electron–phonon scattering rates, as well as the treatment of IPP and impurity scattering. Finally, Section 5, Section 6 and Section 7 present and analyze our transport and FET results.

## 2. Device Geometry and Monte Carlo Simulation Framework

**Figure 1:**
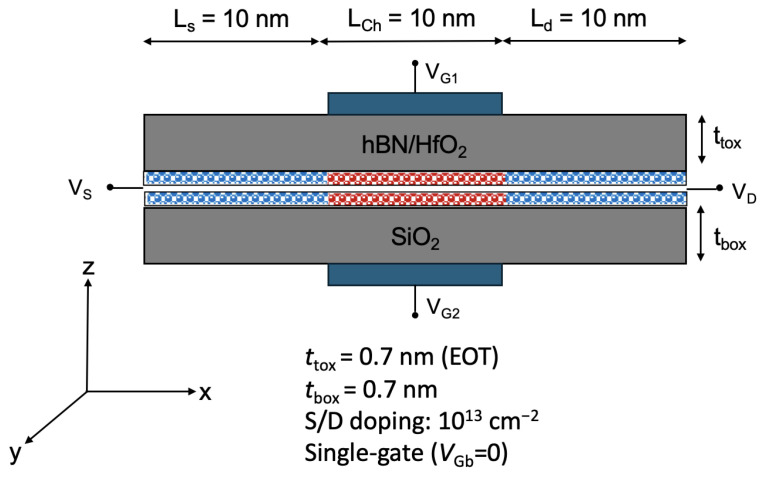
Cross-section of the double-gate bilayer TMD FET considered in this work. The top oxide thickness is denoted by $t_{\mathrm{tox}}$ and the bottom oxide thickness by $t_{\mathrm{box}}$, each corresponding to an equivalent oxide thickness (EOT) of 0.7 nm. The source/drain extension doping concentration is represented as S/D doping. $L_{s}$, $L_{ch}$, and $L_{d}$ refer to the source extension length, channel length, and drain extension length, respectively.

In our work, we have considered a double-gate field-effect transistor (FET) structure with a bilayer TMD as the channel material, shown in Figure 1. The device has a $L_{\mathrm{ch}}=10$ nm channel, with source and drain extensions of equal length $L_{S/D}=10$ nm. The source/drain extensions are doped, while the channel is left undoped. A self-aligned $L_{g}=10$ nm gate is centered on the channel.

The top and bottom gate insulators each result in an equivalent oxide thickness (EOT) of 0.7 nm. This double-gate structure captures the impact of the dielectric environment (substrate and top oxide) on carrier transport, including interface phonons, interface plasmon–phonon (IPP) modes (often improperly called ”remote phonons”), and dielectric screening induced by free carriers and by the environment.

Electronic transport is treated by solving the two-dimensional Boltzmann transport equation (BTE) self-consistently with the Poisson equation using an ensemble Monte Carlo (MC) method. The BTE describes how the carrier distribution evolves over time in real and momentum space under electric fields and scattering. We assume the device to be infinitely wide, such that the gate is much wider than the channel length; this allows us to solve the 2D Poisson equation and neglect edge effects.

The electrostatic potential is obtained by solving the 2D Poisson equation, which accounts for charge distribution and material permittivity. This solution updates the electric field profile used for carrier trajectories in the Monte Carlo simulation. The spatial domain is discretized using non-uniform meshes: one along the transport direction (*x*) and another along the out-of-plane direction (*z*), which is refined near the TMD and oxide interfaces. The Poisson equation is solved using the finite element method adapted from [26] with the following form:

(1) $$\nabla \cdot D\left(r\right)=-\nabla \cdot \left[\varepsilon \left(r\right)\cdot \nabla V\left(r\right)\right]=\rho \left(r\right)=e\left[N_{D}\left(r\right)-n_{\mathrm{el}}\left(r\right)\right],$$

where V(r) is the electrostatic potential, ρ(r) is the charge density, $N_{D}$ is the doping profile, and $n_{\mathrm{el}}$ is the electron density. The dielectric tensor is defined piecewise for each element. In the TMD, we use an anisotropic dielectric tensor:

(2) $$\varepsilon_{2D}=\left[\begin{array}{cc} \varepsilon_{2D_{\|}} & 0\\ 0 & \varepsilon_{2D_{⊥}} \end{array}\right],$$

where $\varepsilon_{2D_{\|}}$ and $\varepsilon_{2D_{⊥}}$ are the in-plane and out-of-plane dielectric constants, respectively. In contrast, the oxide layers use isotropic dielectric constants. The TMD is discretized along *z* using two mesh elements, each of thickness Δz=d/2, where *d* is the total bilayer thickness. The cloud-in-cell method [27] is employed to map the charge of Monte Carlo particles onto the grid nodes at the center of the TMD layer. Dirichlet boundary conditions are assumed at the source and drain terminals, while Neumann conditions are applied elsewhere.

The MC algorithm alternates between two steps: (i) carriers drift through the device under the electric field, and (ii) scattering events are considered based on pre-tabulated rates, which alter energy, momentum, or direction. Carrier positions are then updated, the charge density is recalculated, and the Poisson equation is solved again. Convergence is reached once steady-state transport is established, typically by tracking 2000–5000 carriers over several picoseconds to obtain statistically reliable current–voltage and field–energy characteristics. After each MC iteration and before the next free flight, we record carrier energy, velocity, current, charge density, and electrostatic potential. These are ensemble-averaged over the total simulation time:

(3) $$\langle A\rangle =\frac{1}{\tau_{\mathrm{sim}}}\sum_{i}\tau_{i}{\langle A\rangle }_{\tau_{i}},$$

where $\tau_{\mathrm{sim}}$ is the total simulation time and $\tau_{i}$ the *i*-th free flight duration. In the low-field regime, mobility is extracted from the diffusion constant $D_{\theta }$ using the Einstein relation in the non-degenerate limit:

(4) $$D_{\theta }=\frac{1}{2}\frac{d}{dt}\langle {\left(x_{\theta }-\langle x_{\theta }\rangle \right)}^{2}\rangle ,\;\mu_{\theta }=\frac{eD_{\theta }}{k_{B}T},$$

where $x_{\theta }$ is the carrier position along the transport direction θ. This approach minimizes stochastic noise when the drift velocity is much smaller than the thermal velocity. At high fields, the steady-state drift velocity, carrier energy, and field-dependent mobility are computed as

(5) $$v_{\mathrm{drift}}=\frac{1}{N_{p}}\sum_{i}v_{i},\;\langle E\rangle =\frac{1}{N_{p}}\sum_{i}E_{i},$$

where $N_{p}$ is the total number of simulated carriers, $v_{i}$ is the component of the velocity along transport direction (x-axis), and $E_{i}$ is the kinetic energy. Device-level simulations also yield drain current and spatial profiles of charge density, potential, electric field, and carrier energy for each gate and drain bias point. These data allow us to construct *I*–*V* characteristics and analyze how scattering mechanisms affect carriers across the device.

We perform density functional theory (DFT) calculations to obtain the bilayer TMD band structure and phonon dispersion. The band structure reveals key electronic properties, such as effective masses and valley configurations that govern carrier kinematics, while the phonon dispersion characterizes the lattice vibrations responsible for intrinsic scattering processes. From these fundamental quantities, we tabulate the rates for electron scattering with the TMD phonons, with IPPs, and with ionized impurities (dopant atoms). The following sections provide details on the scattering mechanisms considered in these simulations.

## 3. Band Structure and Phonon Spectrum

**Figure 2:**
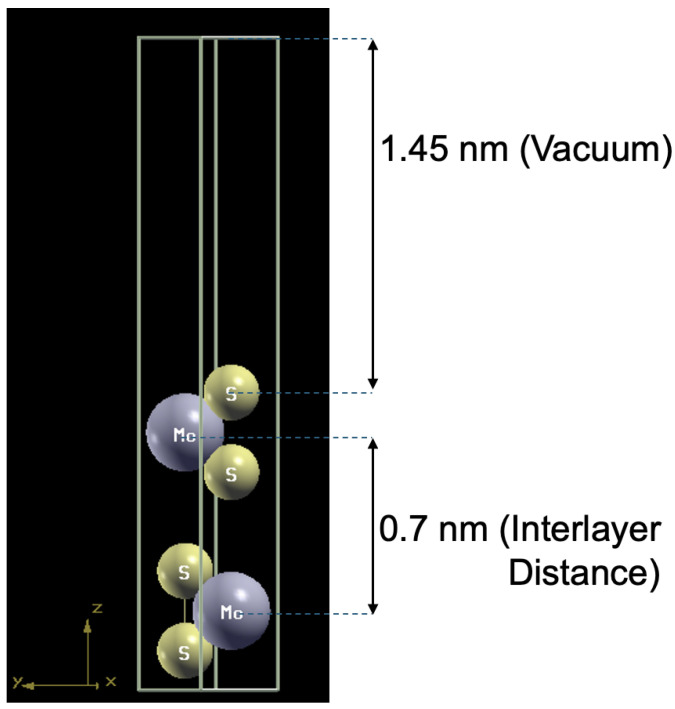
Relaxed atomic structure of bilayer MoS_2_.

Band structures of freestanding bilayer TMDs were calculated using density functional theory (DFT) as implemented in Quantum ESPRESSO [20,21]. We employed the Optimized Norm Conserving Vanderbilt (ONCV) pseudopotentials [28] and GGA-PBE exchange-correlation functional [29,30]. The structures were relaxed until atomic forces were below $10^{-4}$ eV/nm, ensuring energy minimization (Figure 2). A vacuum spacing of approximately 1.4 nm was used between supercells to prevent unphysical interlayer interactions.

The phonon dispersions were computed using density functional perturbation theory (DFPT) [31] with a coarse k-point mesh of 12×12×1 and q-point mesh of 6×6×1, which were verified to give converged total energies with no appreciable change upon further mesh refinement. To obtain accurate phonon energies, especially for low-energy acoustic modes, Wannier interpolation was performed on a finer 30×30×1 mesh using the EPW package [22,23,24]. Spin–orbit coupling (SOC) was included in all calculations as it influences the electronic structure and carrier mobility. The DFT input parameters are summarized in Table 1.

**Figure 3:**
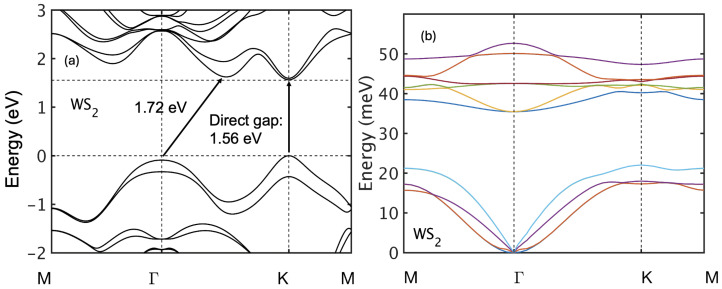
(**a**) Band structure and (**b**) phonon dispersion of bilayer WS_2_.

Figure 3 shows the calculated band structure and phonon dispersion of bilayer WS_2_ along the high-symmetry path M-Γ-K-M. Bilayer WS_2_ exhibits a direct bandgap of 1.56 eV with the conduction band minimum at the K-point and a satellite conduction valley at Q, 58 meV higher in energy. The electron effective masses are isotropic: 0.3 $m_{e}$ at K and 0.74 $m_{e}$ at Q. The valence band maximum lies at K with a local maximum at Γ 87 meV lower, with hole effective masses 0.27 $m_{h}$ at K and 2.1 $m_{h}$ at Γ.

**Figure 4:**
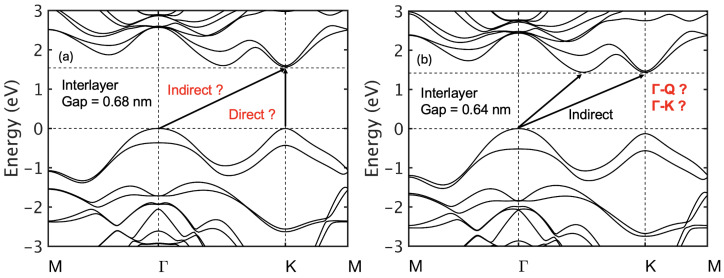
Effect of interlayer distance on bilayer WS_2_ band structure: (**a**) 0.68 nm, (**b**) 0.64 nm. For smaller interlayer gaps, $\Gamma_{v}$ raises while $Q_{c}$ drops. The gap changes from direct K–K to indirect Γ–K and then to indirect Γ–Q with a further reduction in the interlayer gap.

The previous literature reports bilayer WS_2_ as an indirect-bandgap material with the conduction band minimum (CBM) at Γ. We observed, however, that the bandgap character strongly depends on the interlayer spacing (Figure 4). At an interlayer distance of 0.68 nm, the CBM shifts to Γ, producing an indirect gap. Reducing the spacing to 0.64 nm lowers the *Q* valley relative to *K*, restoring a direct gap between *Q* and Γ. This sensitivity of valley ordering to interlayer distance highlights the delicate balance between competing conduction-band valleys in bilayer WS_2_. Even small structural changes, such as strain, alter the nature of the bandgap.

Experimentally, angle-resolved photoemission spectroscopy (ARPES) can probe the valence bands, but direct identification of the CBM remains challenging. This valley sensitivity has direct consequences for transport, as different conduction-band minima (K, Q, or Γ) result in varying effective masses, density of states, and intervalley scattering, which can markedly influence carrier mobility and device performance.

## 4. Scattering Mechanisms

### 4.1. Electron–Phonon Scattering

**Figure 5:**
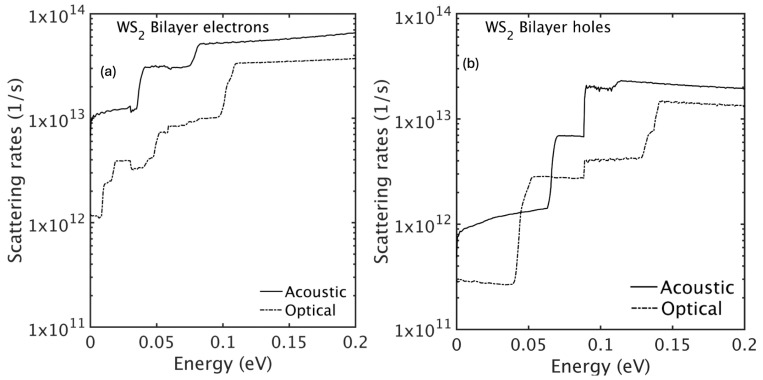
Phonon-limited scattering rates as a function of kinetic energy for (**a**) electrons and (**b**) holes in bilayer WS_2_. The rates are averaged over equi-energy surfaces, with contributions from acoustic and optical phonons shown separately.

Intrinsic electron–phonon scattering is included using first-order perturbation theory. The matrix element for a transition from band *m* at wavevector k to band *n* at k+q via phonon mode ν is

(6) $$g_{mn}^{\nu }\left(k,q\right)=\sqrt{\frac{\hbar }{2\;\omega_{q\nu }}}\langle u_{n,k+q}|\Delta V_{q\nu }^{\mathrm{SCF}}|u_{m,k}\rangle ,$$

where *ℏ* is the reduced Planck constant, $\omega_{q\nu }$ is the frequency of phonon mode ν at wavevector q, and $\Delta V_{q\nu }^{\mathrm{SCF}}$ is the change in the self-consistent potential due to the phonon. The electronic eigenstates $|u_{n,k}\rangle$ are used in evaluating the matrix elements. Matrix elements are computed using density functional perturbation theory (DFPT) and interpolated using maximally localized Wannier functions (MLWFs) via EPW [22,23,24]. The scattering rate is obtained using Fermi’s golden rule:

(7) $$\frac{1}{\tau^{\left(\nu \right)}\left(k,n\right)}=\frac{2\pi }{\hbar }\sum_{k^{\prime },n^{\prime }}{\left|g_{mn}^{\nu }\left(k,q\right)\right|}^{2}\left(N_{q\nu }+\frac{1}{2}\pm \frac{1}{2}\right)\delta \left(E_{n^{\prime }}\left(k^{\prime }\right)-E_{n}\left(k\right)\pm \hbar \omega_{q\nu }\right),\;$$

where $\tau^{\left(\nu \right)}\left(k,n\right)$ is the carrier lifetime for phonon mode ν, $g_{mn}^{\nu }\left(k,q\right)$ is the electron–phonon coupling matrix element, $N_{q\nu }$ is the Bose–Einstein occupation number representing phonon population, and $E_{n}\left(k\right)$ denotes the energy of the electronic state in band *n* at wavevector k. In all our calculations, scattering with out-of-plane acoustic (ZA) phonons is neglected because 2H-phase TMDs possess horizontal mirror ($\sigma_{h}$) symmetry, which forbids first-order carrier–ZA interactions [32]. Higher-order (two-phonon) processes are much weaker in these materials and therefore contribute negligibly to carrier scattering [33]. The sum over final states $\left(k^{\prime },n^{\prime }\right)$ accounts for all possible scattering processes to different bands and wavevectors. The final-state density of states is evaluated using a 2D Gilat–Raubenheimer scheme [34,35]. Energy-resolved scattering rates are obtained by summing the contributions from all electronic states within each energy bin and normalizing by the density of states, yielding average rates that are further separated into acoustic and optical phonon contributions. Figure 5 presents phonon-limited scattering rates for electrons (a) and holes (b) in bilayer WS_2_, showing the contributions of acoustic and optical phonon modes plotted against carrier energy.

### 4.2. Dielectric Screening

The top and bottom dielectrics (*dielectric environment*) screen the electron–phonon interaction [36] and a full DFT calculation of the electron–phonon matrix elements for the entire double-gate structure would require an impractically large supercell. To circumvent this, we employ a semi-empirical approach [36]: the matrix elements are first computed for a free-standing 2D layer using Quantum ESPRESSO and EPW. Since the Hartree term dominates the scattering potential, the free-standing Green’s function (implicitly used in DFT) is replaced with one that satisfies the boundary conditions of the double-gate structure, including both dielectrics. This modification rescales the DFT scattering rates by the squared ratio of the two Green’s functions:

(8) $${\left|\frac{G_{Q,\omega_{Q}^{\left(\eta \right)}}^{\left(\mathrm{env}\right)}\left(d,d\right)}{G_{Q}^{\left(\mathrm{vac}\right)}\left(d,d\right)}\right|}^{2},$$

where *d* is the position at the center of the layer (along the *z*-direction normal to the plane). Here, $G_{Q,\omega_{Q}^{\left(\eta \right)}}^{\left(\mathrm{env}\right)}\left(d,d\right)$ is the Green’s function of the Poisson equation evaluated at z=d for the dielectric environment of the double-gate geometry, at wavevector *Q* and phonon frequency $\omega_{Q}^{\left(\eta \right)}$. $G_{Q}^{\left(\mathrm{vac}\right)}\left(d,d\right)$ is the corresponding Green’s function for the free-standing layer. The Green’s function $G_{Q,\omega_{Q}^{\left(\eta \right)}}^{\left(\mathrm{env}\right)}\left(d,d\right)$ incorporates the effect of the 2D electron gas (2DEG) in the TMD layer, so the procedure described also captures free-carrier screening. A thorough discussion of the assumptions behind the scaling factor in Equation (8) is provided in [36]. Implemented in the Monte Carlo simulations via the rejection method, this rescaling reduces the scattering rates relative to the free-standing case, leading to higher carrier mobility compared to the freestanding layer.

### 4.3. Interface Plasmon–Phonon (IPP) Scattering

The plasma oscillations of electrons in the bilayer couple with the optical phonons of the top-gate dielectric, the substrate, and the polar 2D layer, giving rise to hybrid interface plasmon–phonon (IPP) modes. This hybridization is particularly important as it significantly affects the scattering rates, especially at small momentum transfer *Q*. The full theoretical framework is presented in [36,37], which provides all necessary details; here, we adopt the notation from [36] and summarize the main results for convenience. Because electron–IPP interactions transfer momentum only through the phonon-like content of each hybrid mode, we must evaluate the phonon content $\Phi^{\left(\alpha \right)}\left(\omega_{Q}^{\left(i\right)}\right)$ of phonon α (TO1, TO2, TO3, TO4, or ZO) for every mode *i* [38,39]. In terms of this quantity, the scattering potential amplitude $A_{Q,\omega_{Q}^{\left(i\right)}}^{\left(\alpha \right)}$ for phonon α of mode *i* is given by [36]:

(9) AQ,ωQ(i)(α)2=Φ(α)(ωQ(i))1−e−2Qtb2  e2ℏωQ(i)2Q1ϵTOT(α,high)(Q,ωQ(i))−1ϵTOT(α,low)(Q,ωQ(i)).

In Equation (9), $\epsilon_{\mathrm{TOT}}\left(Q,\omega \right)$ is the dielectric function of the system. The superscripts *high* and *low* correspond to the absence and presence, respectively, of the response from phonon α, allowing us to isolate its contribution to the Fröhlich-like potential. A complete derivation of Equation (9) and the resulting scattering rates can be found in Ref. [36].

As discussed in Ref. [36], scattering with interface hybrid excitations controls carrier transport in high-κ environments. In such dielectrics, this mechanism counteracts the benefits of screening and suppresses the mobility of the 2D channel, often below that of the free-standing case. The reduction becomes more severe as the dielectric constant of the top-gate insulator increases, e.g., when replacing hBN with ZrO_2_.

### 4.4. Landau Damping

**Figure 6:**
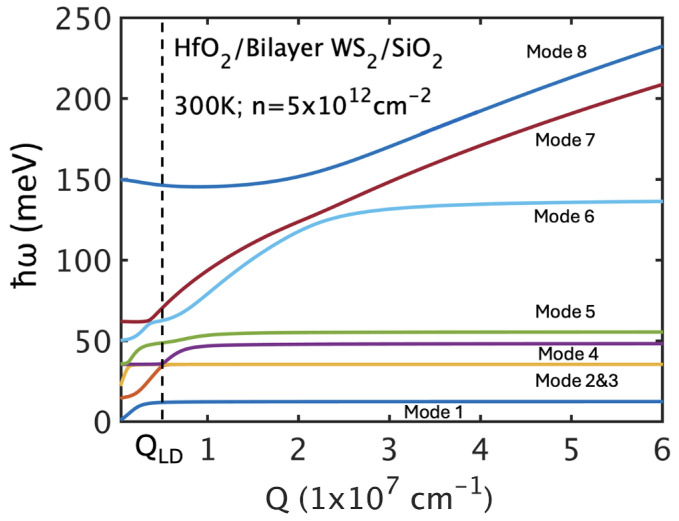
Dispersion of fully hybridized IPP modes for SiO_2_/MoS_2_/HfO_2_ showing Landau damping cutoff wave vector $Q_{LD}$.

Landau damping occurs when plasmons fall inside the single-particle excitation continuum, i.e., $E\left(k_{F}-Q\right)-E\left(k_{F}\right)\leq \hbar \omega_{P}\left(Q\right)\leq E\left(k_{F}+Q\right)-E\left(k_{F}\right)$ ($k_{F}$ being the Fermi wave vector), so that they are no longer well-defined eigenmodes of the system and decay into incoherent electron–hole excitations. The dielectric response of the 2D TMD monolayer is described by

(10) $$\varepsilon_{2D⊥}\left(Q,\omega \right)=\varepsilon_{2D⊥}^{\left(\infty \right)}\;\left[1-e^{2}G_{Q}\left(h/2,h/2\right)\;\Pi_{2D}\left(Q,\omega \right)\right]+\left(\varepsilon_{2D⊥}^{\left(0\right)}-\varepsilon_{2D⊥}^{\left(\infty \right)}\right)\frac{\omega_{ZO}^{2}}{\omega_{ZO}^{2}-\omega^{2}},$$

where $\varepsilon_{2D⊥}^{\left(0\right)}$ and $\varepsilon_{2D⊥}^{\left(\infty \right)}$ are the static and optical out-of-plane dielectric constants of the monolayer, *h* is the effective thickness, $G_{Q}\left(z,z^{\prime }\right)$ is the in-plane Fourier transform of the Poisson Green’s function of the device geometry (evaluated at $z=z^{\prime }=h/2$), $\Pi_{2D}\left(Q,\omega \right)$ is the 2D carrier polarizability given by Stern [40], and $\omega_{ZO}$ is the frequency of the out-of-plane optical (ZO) phonon. In mirror-symmetric (2H) monolayers, the ZO-induced potential is antisymmetric and the electron–phonon matrix element vanishes to first order, so electrons do not couple to this component of the hybrid modes. In the long-wavelength limit (Q→0), the carrier contribution in Equation (10) can be approximated by

(11) $$\varepsilon_{2D}^{\mathrm{el}}\left(Q,\omega \right)\approx \varepsilon_{2D}^{\left(\infty \right)}\;\left(1-\frac{\omega_{P}{\left(Q\right)}^{2}}{\omega^{2}}\right),$$

where the 2D plasmon frequency is defined as $\omega_{P}{\left(Q\right)}^{2}=e^{2}nQ/2\varepsilon_{2D}^{\infty }m^{*}$, with *n* the carrier density and $m^{*}$ the effective mass. The onset of Landau damping is set by the cutoff wave vector ($Q_{LD}$):

(12) $$Q_{LD}={\left[k_{F}^{2}+\frac{2m^{*}}{\hbar^{2}}\;\omega_{P}\left(Q_{LD}\right)\right]}^{1/2}-k_{F},$$

where $k_{F}$ is the Fermi wave vector. For $Q<Q_{LD}$, plasmons remain coherent and dynamically screen the phonon-like hybrid modes; for $Q>Q_{LD}$, we replace the dynamic long-wavelength term $1-\omega_{P}{\left(Q\right)}^{2}/\omega^{2}$ in Equation (11) with the static Thomas–Fermi expression $\varepsilon_{TF}\left(Q\right)=1+Q_{TF}^{2}/Q^{2}$ where $Q_{TF}$ is the screening wave vector give by

(13) $$Q_{TF}=\frac{e^{2}n}{2\varepsilon_{2D}k_{B}T}\;\left(\mathrm{non-degenerate}\right),\;Q_{TF}=\frac{e^{2}m^{*}g}{2\pi \hbar^{2}\varepsilon_{2D}}\;\left(\mathrm{degenerate}\right),$$

with *g* the valley degeneracy. This framework captures the crossover correctly: Below $Q_{LD}$, plasmons dynamically hybridize with phonon-like modes, whereas above $Q_{LD}$, only statically screened phonon-like branches survive. We consider two double-gate dielectric configurations—hBN/bilayer TMD/SiO_2_ (low-κ) and HfO_2_/bilayer TMD/SiO_2_ (high-κ)—to capture the range of remote phonon scattering effects expected in realistic device environments. Figure 6 shows the dispersion of fully hybridized IPP modes in the SiO_2_/WS_2_/HfO_2_ stack, revealing strong phonon–plasmon coupling at small wave vectors *Q*. Above the Landau damping cutoff $Q_{LD}$, plasmons are suppressed and only surface optical (SO) phonons remain.

**Figure 7:**
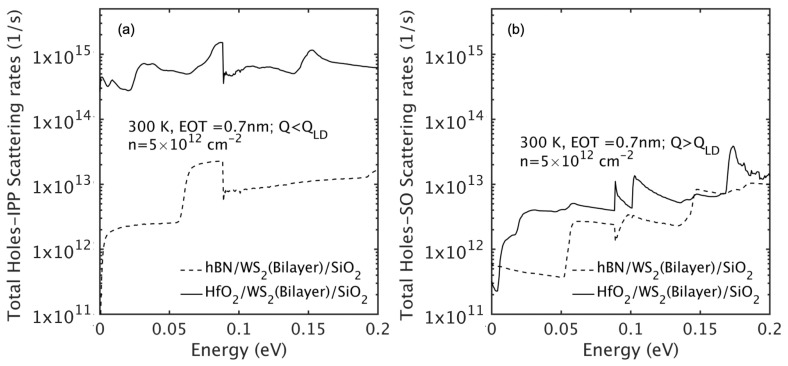
Electron–IPP scattering rates in bilayer WS_2_ for (**a**) $Q\leq Q_{LD}$ (hybridized IPPs) and (**b**) $Q>Q_{LD}$ (surface optical phonons), comparing hBN and HfO_2_ gate dielectrics.

Figure 7 shows the hole-IPP scattering rates separated by wave vector regimes ($Q\leq Q_{LD}$ for hybridized modes and $Q>Q_{LD}$ for decoupled SO modes). Scattering is dominated by long-wavelength hybridized modes, especially in the HfO_2_ configuration due to stronger coupling. In contrast, the hBN stack has weak IPP scattering due to its weak coupling.

Overall, these results underscore the fundamental mobility trade-off when employing high-κ dielectrics, such as HfO_2_: while they provide stronger electrostatic gate control, this advantage is offset by increased remote phonon scattering that degrades carrier mobility. Since IPP scattering rates depend on the local carrier density—which varies spatially within a FET—we pre-tabulate these rates for five carrier densities ranging from $1\times 10^{10}$ to $1\times 10^{14}$ cm^−2^. During the Monte Carlo simulations, the local carrier density at each point is used to interpolate the appropriate IPP scattering rate on a logarithmic scale, ensuring accurate representation of spatially varying IPP rates.

### 4.5. Impurity Scattering and Dielectric Impact

The impact of charged impurities in the TMD channel is evaluated by solving the Poisson Green’s function for the double-gate geometry considered in this work, following Ref. [41]. The corresponding screened Coulomb potential for a charge located at the bilayer center is expressed as

(14) $$\phi_{Q}^{\mathrm{sc}}\left(z=d\right)=\frac{e^{2}G_{Q}^{\left(22\right)}\left(d,d\right)}{1-e^{2}G_{Q}^{\left(22\right)}\left(d,d\right)\Pi_{2D}\left(Q,\omega =0\right)},$$

where Π2D(Q,ω=0) represents the static electronic polarizability of the 2D system, originally formulated by Stern [42] at zero temperature and later generalized to finite temperatures by Maldague [43], and $G^{\left(22\right)}Q\left(d,d\right)$ denotes the Fourier transform of the Poisson Green’s function evaluated with the source charge located at the center of the 2D layer [41]:

(15) $$\Pi_{2D}\left(Q,\omega ;T,E_{F}\right)=\int_{0}^{\infty }d\mu \frac{\Pi_{2D}\left(Q,\omega ;0,\mu \right)}{4k_{B}T\cosh\left(\frac{E_{F}-\mu }{2k_{B}T}\right)}$$

where *T* the temperature, $\Pi_{2D}\left(Q,\omega \right)$ is the zero temperature polarizability, $E_{F}$ the Fermi level and $k_{B}$ is the Boltzmann constant. Lastly, the impurity scattering rate corresponding to an impurity concentration $N_{I}$ can be expressed as

(16) $$\frac{1}{\tau^{\mathrm{imp}}\left(k,n\right)}=\frac{2\pi }{\hbar }N_{I}\sum_{k^{\prime },n^{\prime }}|\langle k^{\prime },n^{\prime }|\phi_{Q}^{\mathrm{sc}}\left(k-k^{\prime }\right){\left|k,n\rangle \right|}^{2}\delta \left(E_{k^{\prime }}-E_{k}\right).$$

**Figure 8:**
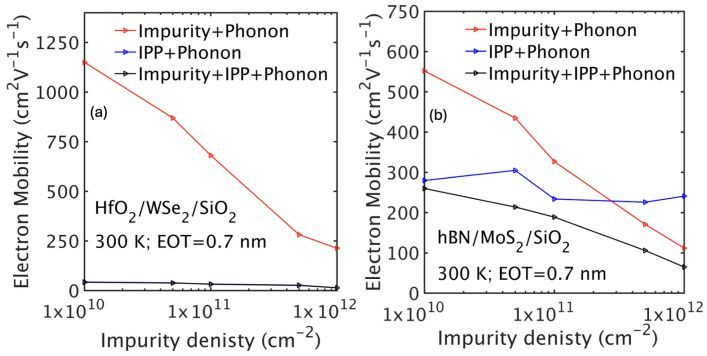
Dependence of electron mobility on impurity density, including different scattering mechanisms for the HfO_2_/WSe_2_/SiO_2_ (**a**) and hBN/MoS_2_/SiO_2_ (**b**) stacks.

The impact of impurity scattering is strongly modulated by the choice of gate dielectric. High-κ top oxides, such as HfO_2_, enhance dielectric screening because their large dielectric constant reduces the electric field generated by impurities. This weaker Coulomb field lowers the scattering strength experienced by carriers, thereby improving impurity-limited mobility. However, as discussed in the previous section, a high-κ dielectric environment also strengthens interface plasmon–phonon (IPP) scattering in the channel, which can significantly degrade carrier mobility. In contrast, low-κ dielectrics like hBN do not amplify IPP scattering as strongly, and in such cases, impurity scattering may be the dominant mechanism depending on the carrier density. This interplay between IPP and impurity scattering has been studied previously [44]. Figure 8a, adapted from [44], shows that with a HfO_2_ top-gate insulator, IPP scattering dominates transport across the entire range of impurity concentrations, whereas in the case of a low-κ hBN top insulator, its influence becomes significant only when the impurity density is below the mid-10^11^ cm^−2^ level.

## 5. Electronic Transport in Bilayer Transition Metal Dichalcogenides

This section presents the carrier transport properties of bilayer transition metal dichalcogenides (TMDs). Table 2 and Table 3 report the calculated hole and electron mobilities at a carrier density of $5\times 10^{12}$ cm^−2^ and T=300 K for freestanding bilayers and for double-gated structures with hBN or HfO_2_ as the top dielectric. Previous theoretical reports [45] have predicted a high hole mobility in WSe_2_ and WS_2_. Similarly, in our study, we found particularly high hole mobilities for bilayer WS_2_ and WSe_2_, underscoring their promise for high-performance devices. At the same time, the introduction of a high-κ dielectric such as HfO_2_ significantly reduces the mobility due to enhanced IPP scattering, whereas low-κ dielectrics like hBN preserve much of the intrinsic transport advantage. A more detailed discussion of these trends is provided below.

Freestanding bilayers exhibit the highest mobilities because carriers scatter only with intrinsic acoustic and optical phonons. For example, we calculate hole and electron mobilities of 2300 cm^2^/Vs and 161 cm^2^/Vs for bilayer WS_2_, and 1300 cm^2^/Vs and 201 cm^2^/Vs for bilayer WSe_2_, respectively.

When hBN is introduced as the top-gate dielectric, mobilities remain close to or in some cases even exceed those of the freestanding case. For instance, the hole mobility in bilayer WSe_2_ increases from 1300 cm^2^/Vs to 1500 cm^2^/Vs, while the electron mobility in WS_2_ improves slightly from 161 cm^2^/Vs to 172 cm^2^/Vs. This beneficial behavior stems from two factors. First, hBN exhibits inherently weak interface plasmon–phonon (IPP) scattering due to its low ionic polarization and the high optical phonon frequencies associated with the light B and N ions. Second, hBN provides additional dielectric screening of Coulomb interactions, which further suppresses scattering from charged impurities and enhances transport characteristics. The combined effect of weak IPP coupling and efficient dielectric screening makes hBN an exceptionally favorable dielectric environment for TMD devices [39].

In contrast, HfO_2_ leads to strong degradation in mobility across all TMDs studied. For WS_2_, the hole mobility drops from 2300 cm^2^/Vs in the freestanding configuration to only 100 cm^2^/Vs, and for WSe_2_, the electron mobility falls from 201 cm^2^/Vs to 36 cm^2^/Vs. This sharp reduction is due to the high dielectric constant and strong ionic polarization of HfO_2_, which significantly enhances remote phonon and IPP scattering. Although the high-κ oxide provides strong electrostatic control, the additional scattering channels dominate, severely limiting mobility. These findings are consistent with earlier reports [38,46] for TMDs, showing that mobility decreases nearly monotonically as the gate insulator’s dielectric constant increases. While hBN offers the best transport performance and can even outperform the freestanding case in some configurations, it comes with trade-offs such as a low dielectric constant that limits gate capacitance and a relatively small barrier height that could increase gate leakage currents if used alone as a gate dielectric.

## 6. Velocity-Field Characteristics

**Figure 9:**
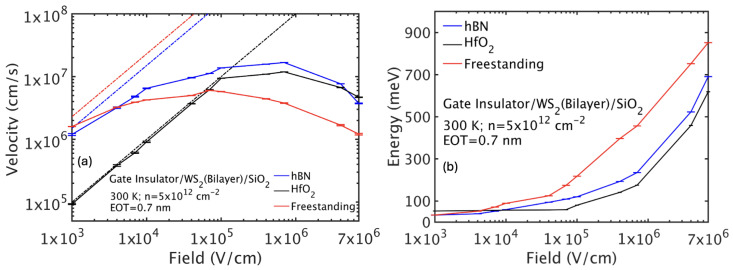
(**a**) Hole drift velocity-field and (**b**) average energy-field characteristics for freestanding and double-gated bilayer WS_2_. To identify the Ohmic region, the dashed lines are drawn by multiplying the low-field mobility with the applied electric field. The results have been obtained assuming oxides with an EOT of 0.7 nm and a carrier sheet density of $5\times 10^{12}$ cm^−2^.

**Figure 10:**
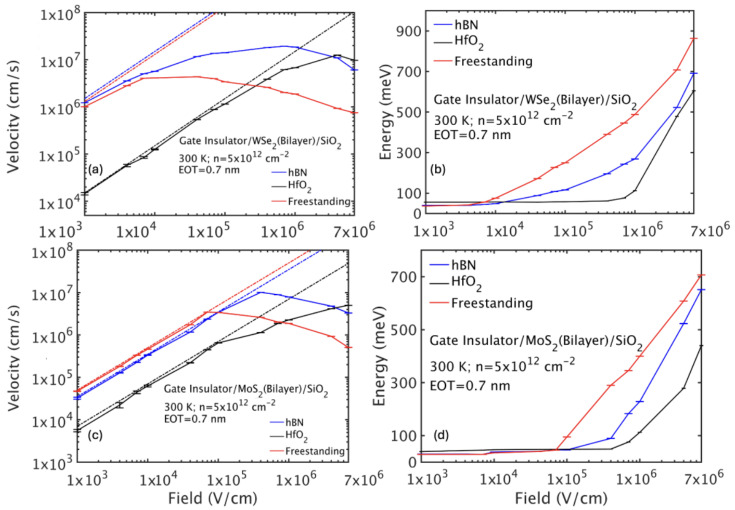
Hole drift velocity-field and average energy-field characteristics for bilayer WSe_2_ (**a**,**b**) and MoS_2_ (**c**,**d**) for free-standing and double-gated layers. To identify the Ohmic region, the dashed lines are drawn by multiplying the low-field mobility with the applied electric field. The results have been obtained assuming oxides with an EOT of 0.7 nm and a carrier sheet density of $5\times 10^{12}$ cm^−2^.

Next, we studied high-field carrier transport for freestanding and double-gated bilayer TMDs (Figure 9 and Figure 10). The drift velocity initially increases linearly with field (Ohmic regime), saturates, and then exhibits negative differential mobility (NDM) at high fields due to carriers populating satellite valleys with heavier effective masses.

For freestanding WS_2_, NDM occurs near $10^{5}$ V/cm as holes gain energy to populate the Γ valley with larger effective mass, reducing drift velocity. In HfO_2_/bilayer WS_2_/SiO_2_, increased scattering suppresses energy gain, shifting NDM onset to fields above $5\times 10^{6}$ V/cm. The hBN configuration behaves similarly to freestanding, but with slightly delayed NDM.

Bilayer WSe_2_ exhibits a similar behavior with NDM onset around $10^{4}$ V/cm in freestanding conditions but delayed under HfO_2_. Bilayer MoS_2_ has lower mobility and delayed NDM onset due to stronger intrinsic scattering and dielectric effects, with NDM absent in the HfO_2_ environment over the field range studied.

In summary, bilayer TMDs exhibit transport strongly modulated by the dielectric environment. Freestanding layers achieve the highest mobilities, limited only by intrinsic phonon scattering. Low-κ dielectrics like hBN moderately reduce mobility by remote phonon scattering, whereas high-κ materials such as HfO_2_ drastically degrade mobility due to enhanced interface plasmon–phonon scattering. High-field transport shows NDM related to satellite valley population, which is suppressed by increased scattering in high-κ stacks. These findings emphasize the critical trade-offs in device design between gate control and mobility retention in bilayer TMD-based transistors.

From the mobility results, it is evident that bilayer WSe_2_ and WS_2_ are strong candidates for future FET technologies. Compared to their monolayer counterparts, the bilayers generally exhibit higher hole mobilities (e.g., bilayer WS_2_: 2300 cm^2^/Vs vs. monolayer WS_2_: 750 cm^2^/Vs) but similar electron mobilities.

## 7. Bilayer TMD-Based Double-Gate MOSFETs

In this section, we analyze the performance of double-gate MOSFET architectures employing TMD bilayers as channels, focusing on how design choices affect electrostatic control, scattering, and series resistance. As device dimensions scale aggressively into the sub-10 nm regime, maintaining strong gate control is critical. To address this, the semiconductor industry has adopted high-κ dielectrics such as HfO_2_, which enable reduced equivalent oxide thickness (EOT) while mitigating gate leakage. While hBN provides low scattering and yields high mobilities, its relatively low dielectric constant makes it less attractive for scaled CMOS technologies. Motivated by this tradeoff, we concentrate on evaluating device performance with HfO_2_ as the top-gate dielectric, assessing its impact on transport and electrostatics in bilayer TMD channels.

Our first goal is to identify the most promising device architecture. We begin with a 30 nm long double-gate nFET based on bilayer WSe_2_ and HfO_2_ as the top oxide. In our discussions, we will first study the electrostatics of this baseline device using phonon-limited transport. For the final device design, we include the complete scattering set (phonon, interface plasmon–phonon, impurity) and evaluate multiple configurations involving HfO_2_ and hBN as top oxides, and bilayer WSe_2_ and WS_2_ as channels for both nFET and pFET.

The first design is a double-gate n-type MOSFET with bilayer WSe_2_ as the channel and HfO_2_ as the top gate oxide, shown in Figure 1. The device has a channel length of $L_{\mathrm{ch}}=10$ nm and source/drain extensions of $L_{S/D}=10$ nm. The channel is intrinsic, and the source/drain regions are degenerately doped. A self-aligned top gate of $L_{g}=10$ nm is centered over the channel. The EOT is given by:

$$\epsilon_{{\mathrm{SiO}}_{2}}\cdot t_{{\mathrm{HfO}}_{2}}=\epsilon_{{\mathrm{HfO}}_{2}}\cdot t_{{\mathrm{SiO}}_{2}}$$

**Figure 11:**
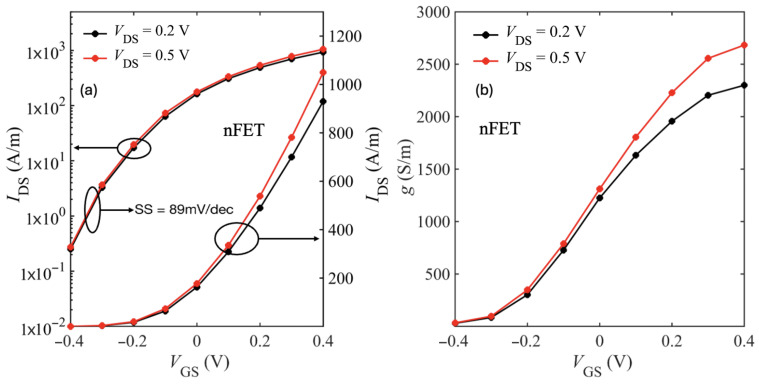
Transfer characteristics ($I_{\mathrm{DS}}$–$V_{\mathrm{GS}}$) on linear and logarithmic scale (**a**) and the corresponding transconductance (**b**) for the HfO_2_/bilayer WSe_2_/SiO_2_ device ($L_{\mathrm{ch}}=10$ nm, $L_{S/D}=10$ nm).

Assuming a 0.7 nm EOT and using $\epsilon_{{\mathrm{SiO}}_{2}}\approx 3.9$, $\epsilon_{{\mathrm{HfO}}_{2}}\approx 22$, the physical oxide thickness is $t_{{\mathrm{HfO}}_{2}}\approx 4.2$ nm. Figure 11 shows the transfer characteristics at $V_{\mathrm{DS}}=0.2$ V and 0.5 V. The device achieves an on-current $I_{\mathrm{on}}\approx 1000$ A/m at $V_{\mathrm{DS}}=0.5$ V, a threshold voltage $V_{T}\approx -0.05$ V, and a subthreshold slope (SS) of ≈ 89 mV/dec.

In scaled FETs with high-κ dielectrics, electrostatics can be affected by strong fringing fields at the gate edges. These are electric field lines that do not pass vertically through the channel but instead fringe laterally into the source and drain regions. Fringing fields degrade short-channel performance by distorting the potential profile and weakening gate control. TMDs like bilayer WSe_2_ have high out-of-plane permittivity ($\varepsilon_{2D_{⊥}}=12$–15) and low in-plane permittivity ($\varepsilon_{2D_{\|}}=5$–7), which amplifies fringing field penetration through the high-κ gate dielectric. As noted in [47,48], this results in stronger short-channel degradation, especially increased DIBL and worse SS.

**Figure 12:**
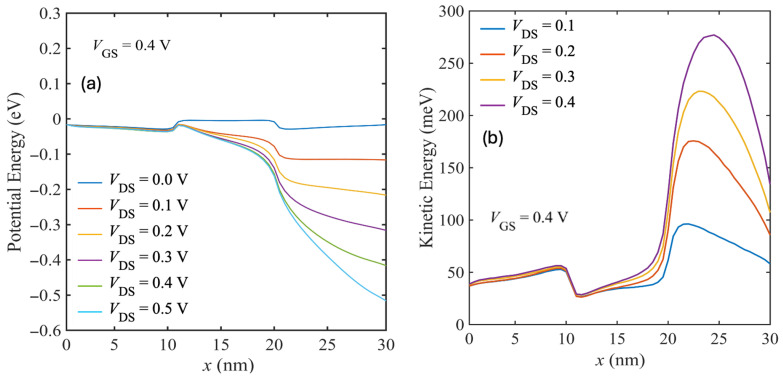
(**a**) Channel potential energy and (**b**) average electron kinetic energy for different $V_{\mathrm{DS}}$ at $V_{\mathrm{GS}}=0.4$ V for the HfO_2_/bilayer WSe_2_/SiO_2_ device.

**Figure 13:**
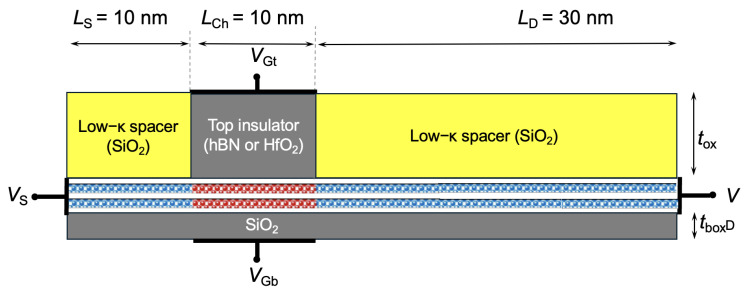
Cross-section of the modified double-gate bilayer WSe_2_ MOSFET with SiO_2_ spacers and a 30 nm drain extension.

Figure 12a shows the potential energy profile along the channel. The steep potential profile near the drain end at higher $V_{\mathrm{DS}}$ corresponds to electric fields that exceed the Ohmic region. In Figure 12b, the average electron kinetic energy increases near the drain for higher biases, exceeding 250 meV at $V_{\mathrm{DS}}=0.4$ V. This corresponds to substantial carrier heating in high-field regions. As discussed in Section 4.3, IPP scattering is very strong in a high-k dielectric environment. But, we need high-κ to maintain the gate control. A solution to mitigate this issue is using low-κ spacer dielectric in the source and drain region, but keeping the high-κ HfO_2_ above the channel region. So, we introduced low-κ SiO_2_ spacers in the source/drain extensions and extended the drain length to 30 nm, Figure 13, to also allow the carriers to cool before reaching the contact.

**Figure 14:**
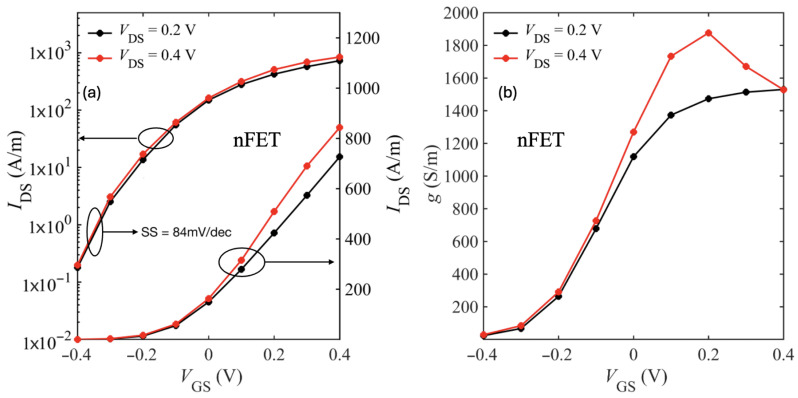
Transfer characteristics ($I_{\mathrm{DS}}$–$V_{\mathrm{GS}}$) (**a**) and transconductance (**b**) for the SiO_2_-HfO_2_-SiO_2_/bilayer WSe_2_/SiO_2_ device device (30 nm drain extension).

Figure 14 shows the transfer characteristics of the large-drain device with spacers and only phonon scattering. We observe marginal improvement in subthreshold swing (SS ≈ 84 mV/dec). However, the extended drain region introduces parasitic resistance that reduces both the ON-current ($I_{\mathrm{on}}$) and transconductance.

**Figure 15:**
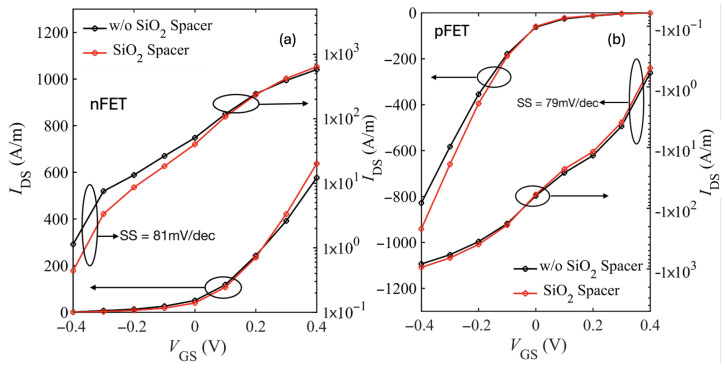
Transfer characteristics ($I_{\mathrm{DS}}$–$V_{\mathrm{GS}}$) nFET (**a**) and pFET (**b**) for the SiO_2_-HfO_2_-SiO_2_/bilayer WSe_2_/SiO_2_ device device (30 nm drain extension).

While these results account only for intrinsic phonon scattering, additional scattering mechanisms such as interface plasmon–phonons are expected to further degrade $I_{\mathrm{on}}$ by increasing the total series resistance. Figure 15 shows the spacers comparison transfer characteristics of the large-drain device with phonon and IPP scattering included. As expected, we see improvement in $I_{\mathrm{on}}$ with the low-k spacers. At low bias, we see higher current for the device without spacers owing to the fringe-induced barrier lowering (FIBL) as discussed in [48].

High $I_{\mathrm{on}}$ remains a critical performance metric for logic transistors, as it directly influences switching speed, drive strength, and energy efficiency. A higher $I_{\mathrm{on}}$ enables faster charging and discharging of load capacitances, reducing gate delay and improving overall circuit throughput. Maintaining high drive current is critical for ensuring fast switching and high performance in scaled logic devices, while also improving power efficiency by reducing switching delays [49,50].

**Figure 16:**
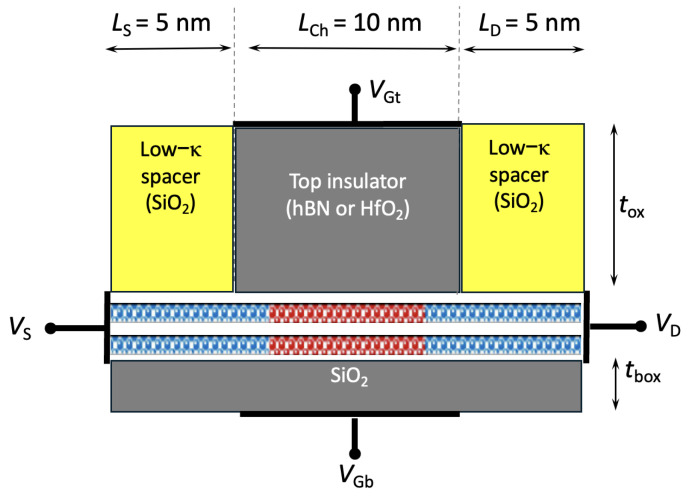
Compact double-gate bilayer WSe_2_ device with 5 nm source/drain extensions.

To improve $I_{\mathrm{on}}$, reducing source/drain extension length is necessary to suppress parasitic resistance, as shown in Figure 16, which illustrates the final device design with shorter source/drain extensions. This design choice, however, comes at the cost of increased hot carrier generation. Hot-carrier degradation in aggressively scaled devices is a well-characterized reliability challenge. However, comprehensive modeling of HCD under various bias conditions enables designers to predict degradation and implement mitigations early in the design process [51,52].

To quantify the impact of various scattering mechanisms on device performance, we simulate pFETs with increasing scattering complexity: starting with only bulk phonons, then adding interface plasmon–phonon (IPP) scattering, and finally including impurity scattering. We use pFETs for this analysis because bilayer WSe_2_ exhibits higher hole mobility, leading to higher baseline Ion values—making the impact of added scattering mechanisms more evident. As shown in Table 4, the long-channel (30 nm drain) pFET suffers a sharp drop in $I_{\mathrm{on}}$ from 950 A/m to 283 A/m as scattering increases. In contrast, a short-channel (5 nm drain) pFET with all scattering mechanisms still delivers a high $I_{\mathrm{on}}$ of 820 A/m, demonstrating that aggressive scaling can recover performance even under significant scattering.

**Figure 17:**
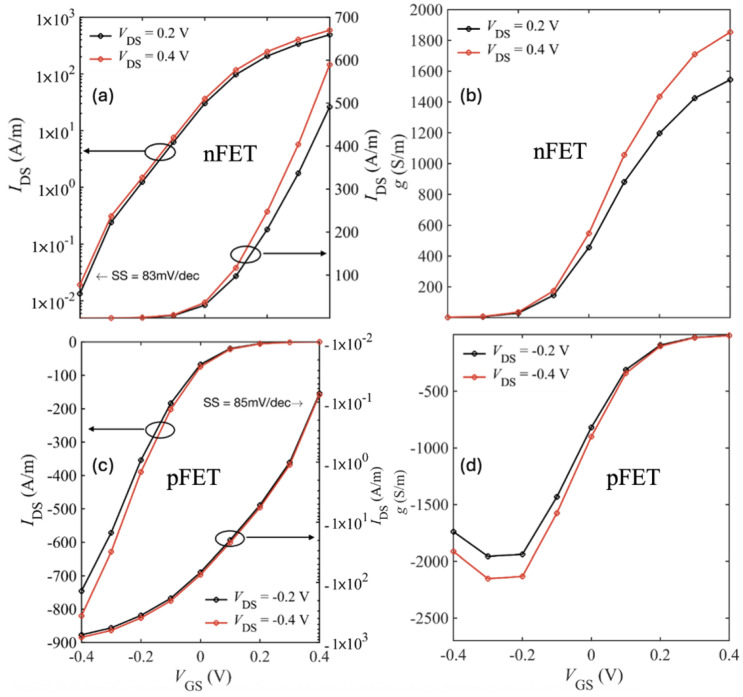
Transfer characteristics ($I_{\mathrm{DS}}$–$V_{\mathrm{GS}}$) on linear and logarithmic scale and the corresponding transconductance for the 5 nm S/D SiO_2_-HfO_2_-SiO_2_/bilayer WSe_2_/SiO_2_ nFET (**a**,**b**) and pFET (**c**,**d**).

**Figure 18:**
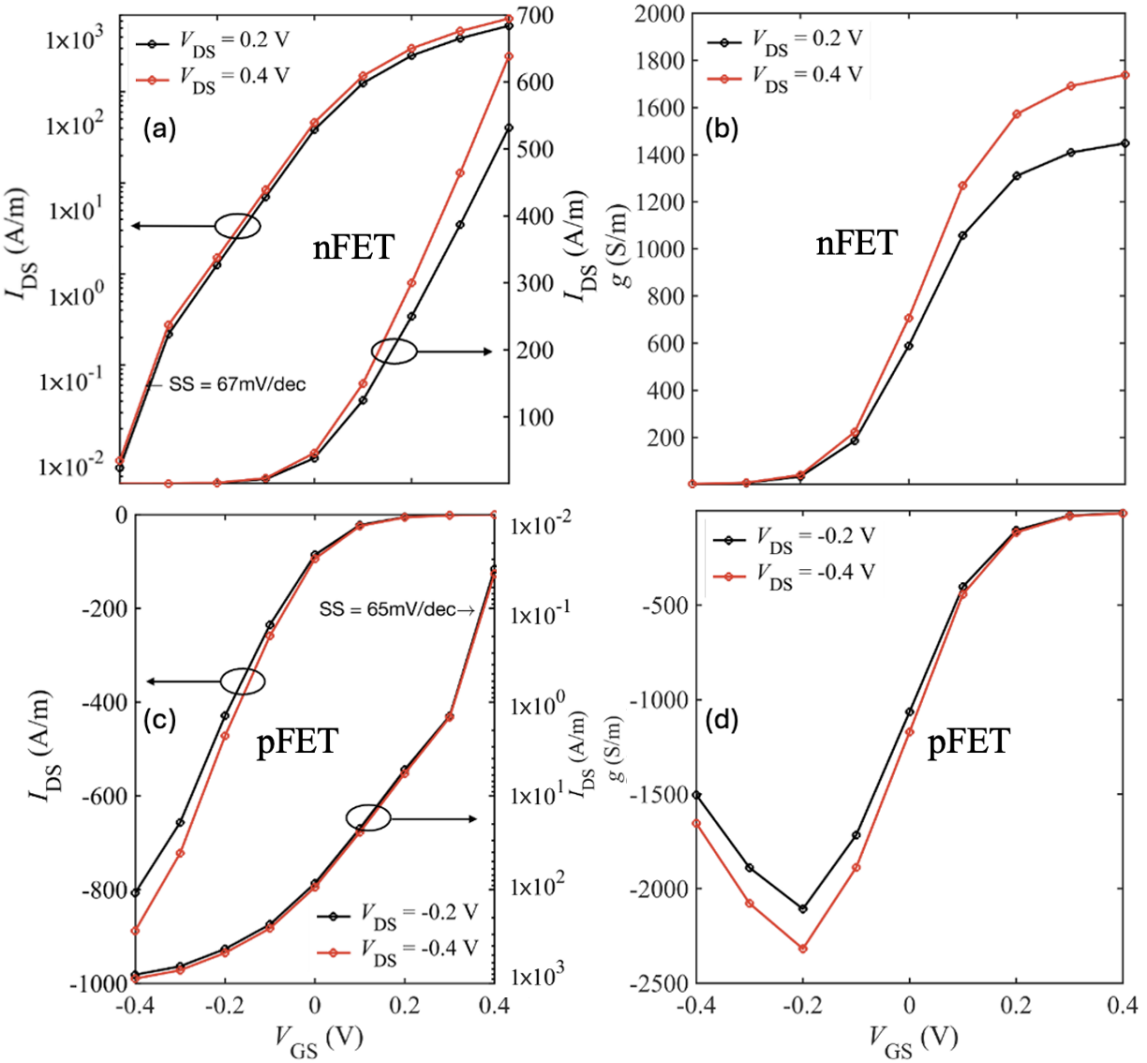
Transfer characteristics ($I_{\mathrm{DS}}$–$V_{\mathrm{GS}}$) on linear and logarithmic scale and the corresponding transconductance for the 5 nm S/D SiO_2_-hBN-SiO_2_/bilayer WSe_2_/SiO_2_ nFET (**a**,**b**) and pFET (**c**,**d**).

Finally, we present all the results for an ultra-short device with 5 nm source and drain extension. Figure 17 and Figure 18 show the *I*–*V* characteristics of bilayer WSe_2_ nFETs and pFETs, comparing devices with hBN and HfO_2_ as top gate dielectrics and SiO_2_ spacers.

**Figure 19:**
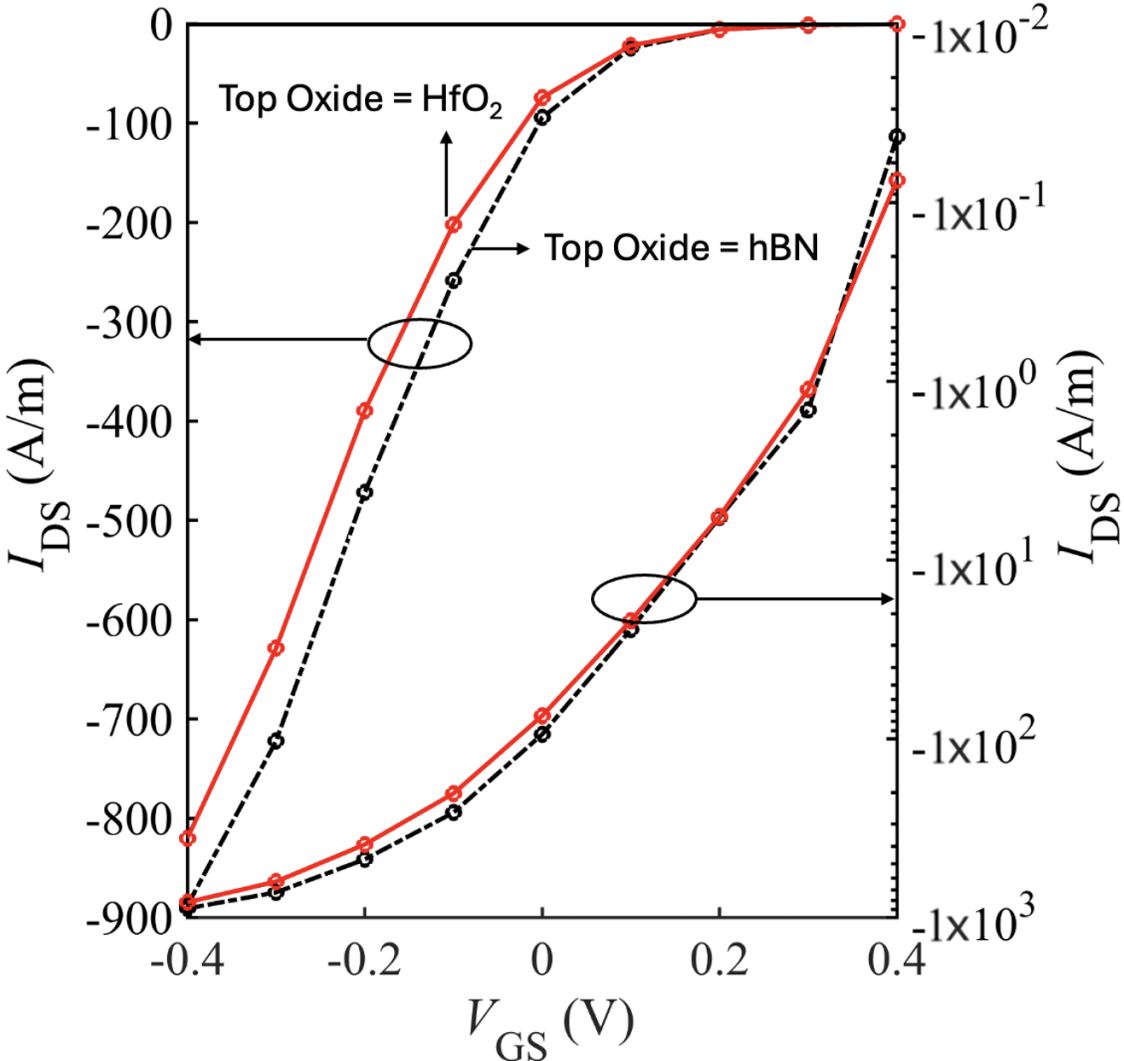
Transfer characteristics ($I_{\mathrm{DS}}$ vs. $I_{\mathrm{GS}}$) for the SiO_2_-HfO_2_-SiO_2_/bilayer-WSe_2_/SiO_2_ pFET with 5 nm S/D extentions, plotted on linear and log scale.

Table 5 and Table 6 summarize the ON-current, transconductance, and SS for short-drain nFET and pFET designs based on bilayer WSe_2_ and WS_2_. Using HfO_2_ as the top gate insulator and including all scattering mechanisms (bulk phonons, IPP, and impurity), the best performance was achieved with the SiO_2_–HfO_2_–SiO_2_/bilayer-WSe_2_/SiO_2_ pFET, which reached $I_{\mathrm{on}}=820$ A/m at $V_{\mathrm{GS}}=V_{\mathrm{DS}}=0.4$ V. Replacing HfO_2_ with hBN as the top oxide further improved $I_{\mathrm{on}}$ by ∼10% to 890 A/m as shown in Figure 19.

## 8. Conclusions

We have presented a comprehensive investigation of carrier transport and device performance in bilayer transition metal dichalcogenides (TMDs), using full-band Monte Carlo simulations. Our study first examined the low-field mobility and high-field velocity-field characteristics for several bilayer TMDs—including WS_2_, WSe_2_, and MoS_2_—in freestanding and double-gated configurations with hBN and HfO_2_ dielectrics.

The results clearly show that the dielectric environment plays a pivotal role in modulating transport. Freestanding bilayers exhibit the highest mobilities, limited only by intrinsic phonon scattering. hBN, due to its weak ionic polarization and high phonon energy, retains high mobility and, in some cases, even improves upon the freestanding case by screening out-of-plane fields while minimally enhancing interface plasmon–phonon (IPP) scattering. In contrast, high-κ materials like HfO_2_ drastically degrade mobility due to strong IPP and remote phonon scattering, despite offering better electrostatic control. These trends were observed consistently across all bilayer TMDs studied, reinforcing the importance of dielectric selection in device design. Our FET simulations indicate that scaling down the source/drain extensions significantly improves the ON current ($I_{\mathrm{on}}$) by reducing series resistance, though care must be taken to manage potential reliability issues such as hot-carrier degradation. High-field transport characteristics reveal strong velocity saturation and negative differential velocity (NDV), especially in freestanding and hBN configurations. These effects are suppressed in high-κ environments due to increased scattering.

By considering HfO_2_ as the top gate dielectric and accounting for bulk phonon, interface plasmon–phonon (IPP), and impurity scattering mechanisms, the optimal device configuration was identified as the SiO_2_-HfO_2_-SiO_2_/bilayer-WSe_2_/SiO_2_ pFET with 5 nm source/drain extensions. This setup achieves an ON current ($I_{\mathrm{ON}}$) of 820 A/m at $V_{\mathrm{GS}}=V_{\mathrm{DS}}=0.4$ V, meeting ITRS performance benchmarks. Replacing the top gate dielectric with hBN leads to a modest increase in $I_{\mathrm{ON}}$ to approximately 890 A/m under the same bias conditions, marking a 10% improvement. These findings highlight the critical role of channel-dielectric engineering and device architecture optimization in enhancing the performance of bilayer TMD-based transistors. While direct quantitative comparison with experiments is challenging due to differences in channel length, gate insulators, and modeling assumptions, single-layer and double-layer devices show comparable performance. The simulated subthreshold swing of 89 mV/dec aligns closely with the 90 mV/dec reported in the computational study of [53] for devices with the same dimensions, although contact resistance may introduce larger differences. Moreover, experimental validation is further complicated by the difficulty of achieving uniform bilayer TMD growth via chemical vapor deposition (CVD), as initiating the second layer is inherently challenging due to the absence of dangling bonds, although recent advances have shown promising progress for MoS_2_, WSe_2_, and WS_2_ [54,55,56].

In summary, bilayer WSe_2_ and WS_2_ could, in principle, enable sub-10 nm FETs that meet the latest ITRS high-performance targets, offering favorable transport and electrostatic characteristics. However, realizing high-performance devices requires careful trade-offs between electrostatic control, mobility degradation, and reliability, as well as attention to fabrication challenges such as high-quality channel growth, gate insulator deposition, and low-resistance contacts. Future extensions of our Monte Carlo framework could explicitly incorporate contact resistance, complementing our present focus on intrinsic device performance, as also explored in related studies [57,58]. Our results underscore the critical role of accurate physical modeling, including scattering mechanisms and dielectric effects, in guiding the optimization of bilayer TMD-based transistors.

## Figures and Tables

**Table 1:** Quantum ESPRESSO computational parameters for bilayer TMD calculations.

Parameter	Value
Kinetic energy cutoff	60 Ry
Ionic minimization threshold	$10^{-6}$ Ry
Self-consistent field threshold	$10^{-12}$ Ry
Charge density cutoff	240 Ry
*k*-point mesh	12×12×1
*q*-point mesh	6×6×1

**Table 2:** Calculated hole mobility (cm^2^V^−1^s^−1^) for different configurations at 300 K.

Material	Freestanding	hBN/TMD/SiO_2_	HfO_2_/TMD/SiO_2_
Bilayer WS_2_	2300	1530	100
Bilayer WSe_2_	1300	1500	13
Bilayer MoS_2_	50	35	7
Monolayer WS_2_	750	1500	70

**Table 3:** Calculated electron mobility (cm^2^V^−1^s^−1^) for different configurations at 300 K.

Material	Freestanding	hBN/TMD/SiO_2_	HfO_2_/TMD/SiO_2_
Bilayer WS_2_	161	172	30
Bilayer WSe_2_	201	163	36
Bilayer MoS_2_	400	340	63
Monolayer WS_2_	170	152	20

**Table 4:** ON-current ($I_{\mathrm{on}}$) in SiO_2_–HfO_2_–SiO_2_/bilayer-WSe_2_/SiO_2_ pFETs for different drain lengths and scattering mechanisms.

Bias Condition	Bulk Phonons (30 nm Drain)	Bulk Phonons + IPP (30 nm Drain)	Bulk Phonons + IPP + Impurity (30 nm Drain)	Bulk Phonons + IPP + Impurity (5 nm Drain)
$V_{\mathrm{GS}}=0.3$ V; $V_{\mathrm{DS}}=0.4$ V	740 A/m	660 A/m	216 A/m	630 A/m
$V_{\mathrm{GS}}=0.4$ V; $V_{\mathrm{DS}}=0.4$ V	950 A/m	870 A/m	283 A/m	820 A/m

**Table 5:** Performance metrics for short-drain WSe_2_ devices ($V_{\mathrm{DS}}=0.4$ V).

Device	$I_{DS}$ (A/m)	$g_{m}$ (S/m)	SS (mV/dec)
nFET, HfO_2_	590	1850	83
nFET, hBN	640	1750	67
pFET, HfO_2_	820	2200	85
pFET, hBN	890	2400	65

**Table 6:** Performance metrics for short-drain WS_2_ devices ($V_{\mathrm{DS}}=0.4$ V).

Device	$I_{\mathrm{DS}}$ (A/m)	$g_{m}$ (S/m)	SS (mV/dec)
nFET, HfO_2_	330	980	90
nFET, hBN	390	1050	73
pFET, HfO_2_	800	2500	89
pFET, hBN	870	2400	70

## Data Availability

Dataset available on request from the authors.
